# How general practitioners would deprescribe in frail oldest-old with polypharmacy — the LESS study

**DOI:** 10.1186/s12875-018-0856-9

**Published:** 2018-10-12

**Authors:** Sophie Mantelli, Katharina Tabea Jungo, Zsofia Rozsnyai, Emily Reeve, Clare H. Luymes, Rosalinde K. E. Poortvliet, Arnaud Chiolero, Nicolas Rodondi, Jacobijn Gussekloo, Sven Streit

**Affiliations:** 1 Institute of Primary Health Care Bern(BIHAM), University of Bern, Mittelstrasse 43, 3012 Bern, Switzerland; 2 NHMRC Cognitive Decline Partnership Centre, Kolling Institute of Medical Research, Northern Clinical School, Faculty of Medicine and Health, University of Sydney, Sydney, Australia; 3 Geriatric Medicine Research, Dalhousie University and Nova Scotia Health Authority, Halifax, NS Canada; 4 College of Pharmacy, Dalhousie University, Halifax, NS Canada; 5 Department of Public Health and Primary Care, Leiden University Medical Center, Leiden, The Netherlands; 6 Department of Epidemiology, Biostatistics and Occupational Health, McGill University, Montreal, Canada; 7 Department of General Internal Medicine, Inselspital, Bern University Hospital, University of Bern, Bern, Switzerland; 8 Department of Internal Medicine, section Gerontology and Geriatrics, Leiden University Medical Center, Leiden, the Netherlands

**Keywords:** Deprescribing, Polypharmacy, Multimorbidity, Old age, Frailty, Complexity

## Abstract

**Background:**

Many oldest-old (> 80-years) with multimorbidity and polypharmacy are at high risk of inappropriate use of medication, but we know little about whether and how GPs would deprescribe, especially in the frail oldest-old. We aimed to determine whether, how, and why Swiss GPs deprescribe for this population.

**Methods:**

GPs took an online survey that presented case-vignettes of a frail oldest-old patient with and without history of cardiovascular disease (CVD) and asked if they would deprescribe any of seven medications. We calculated percentages of GPs willing to deprescribe at least one medication in the case with CVD and compared these with the case without CVD using paired t-tests. We also included open-ended questions to capture reasons for deprescribing and asked which factors could influence their decision to deprescribe by asking for their agreement on a 5-point-Likert-scale.

**Results:**

Of the 282 GPs we invited, 157 (56%) responded: 73% were men; mean age was 56. In the case-vignette without CVD, 98% of GPs deprescribed at least one medication (usually cardiovascular preventive medications) stating it had no indication nor benefit. They would lower the dose or prescribe pain medication as needed to reduce side effects. Their response was much the same when the patient had a history of CVD. GPs reported they were influenced by ‘risk’ and ‘benefit’ of medications, ‘quality of life’, and ‘life expectancy’, and prioritized the patient’s wishes and priorities when deprescribing.

**Conclusion:**

Swiss GPs were willing to deprescribe cardiovascular preventive medication when it lacked indication but tended to retain pain medication. Developing tools for GPs to assist them in balancing the risks and benefits of medication in the context of patient values may improve deprescribing activities in practice.

**Electronic supplementary material:**

The online version of this article (10.1186/s12875-018-0856-9) contains supplementary material, which is available to authorized users.

## Background

General practitioners (GPs) often see oldest-old (> 80 years) and multimorbid patients [1, 2]. Multimorbidity (> 3 chronic conditions) is strongly associated with age and use of multiple medications [3]. In a random sample of Swiss patients [4], 37% of those over 70 took 5 or more medications each day, meeting the common definition of polypharmacy [5]; 44% of patients with polypharmacy took at least one potentially inappropriate medication [4]. Both polypharmacy and inappropriate medication use can increase risk of adverse events in older individuals, including adverse drug events (ADE) [6, 7], medical errors [8], non-compliance, falls [9], impaired physical and cognitive function, hospitalization [10], higher costs of care [11] and mortality [12].

Though these harms are well-established in cross-sectional and longitudinal studies, health care professionals do not have as much clear evidence about either the benefits or safety of stopping or reducing inappropriate medications (deprescribing) [13, 14]. Deprescribing is *‘the process of withdrawal of an inappropriate medication, supervised by a health care professional with the goal of managing polypharmacy and improving outcomes’* [15]. Deprescribing can reduce ADEs and improve patient quality of life and should be integrated into clinical care [16, 17].

Recent reviews show that appropriately planned and monitored deprescribing is feasible and safe [13, 18, 19] but clinicians may be uncomfortable deprescribing for a variety of reasons including fear of unknown negative consequences of change, the existence of other prescribers, and perceived patient/family expectations [20]. GPs also report that the lack of evidence-based clinical practice guidelines can pose a barrier to deprescribing [20–23]. Treatment guidelines rarely discuss when and how medications should be deprescribed or clearly describe appropriate treatment of older adults with multimorbidity and polypharmacy [24–26]. The lack of specific recommendations may be explained by a scarcity of evidence, since older adults with multimorbidity and polypharmacy are often excluded from the randomized controlled trials (RCTs) [27] that inform guideline development.

GPs do have access to various tools that help them identify inappropriate medications or those suitable for deprescribing, such as lists of medications that may be inappropriate for older adults (e.g., the Beers criteria [28] and the STOPPFrail tool [26]), to implicit guides, and overall processes for deprescribing. However, the usefulness (such as the relevance of PIMs lists to complex individuals) and feasibility (e.g. time taken to complete complex review) of these in regular practice has not yet been established [29]. Complex medical, social and ethical situations also make this group harder to treat [30, 31]. Thus, deprescribing in frail oldest-old and multimorbid patients with polypharmacy poses a challenge to GPs that few studies have explored [32].

We used a survey with case-vignettes to determine whether, how and why GPs deprescribe in frail oldest-old patients with multimorbidity and polypharmacy, and to identify factors that influenced their decision to deprescribe.

## Methods

### Design

In the LESS Study (*“Barriers and enab****L****ers to willingn****ES****s to depre****S****cribing in older patients with multimorbidity and polypharmacy and their General Practitioners”)* we report the findings of a cross-sectional survey in Swiss GPs.

### Participants

We set out to sample a group of GPs (*N* = 282) from all regions of Switzerland who had taken part in earlier case-vignette studies and were open to invitations to participate in future research projects [33, 34]. We included all respondents who were currently practicing GPs in Switzerland.

### Survey

We used the same method we employed previously to describe GP decisions about antihypertensive treatment in oldest-old patients [33, 34], and developed an online survey with three sections (A-C): A) GP characteristics and self-reported frequency of deprescribing in oldest-old; B) two case-vignettes of frail oldest-old patients with contrasting histories of CVD; and, C) questions designed to identify factors that may have affected GPs’ decisions to deprescribe. (See Additional file 1 for the complete questionnaire).

For the case-vignettes in part B, our research team, composed mostly of GPs, came to consensus on a scenario that represented a typical patient seen in primary care, and medications frequently prescribed to patients ≥70 years. We generated two fictitious case-vignettes featuring an 82-year-old patient who presented to the GP for a consultation. His frailty was indicated by severely impaired physical and cognitive functions, a last-recorded MMSE of 12/30, his residence in a nursing home, and his complete dependency in activities of daily living [35]. He was being treated with aspirin 100 mg (once daily [od]), atorvastatin 40 mg (od), enalapril 10 mg (od), amlodipine 5 mg (od), paracetamol 1 g (three times daily [tid]), tramadol 50 mg (twice daily [bid]), and pantoprazole 20 mg (od). The survey asked GPs which medications (if any) they would cease or reduce (both covered by the term ‘deprescribing’). The vignettes presented the same patient, but the second added a positive history of CVD. We included an open-ended question where we invited GPs to explain why they chose to deprescribe that medication.

In Part C, we asked GPs to rate the importance of sixteen factors that might influence their decision to deprescribe (5-Point Likert-scales, ranging from “not important” to “very important”). The factors were drawn from the analyses of Anderson et al. [20], and Luymes et al. [36]. An open-ended final question invited participants to name other factors and make additional comments.

To test for clarity and feasibility, we piloted the survey among five experienced GPs. Then sent invitations via email to GPs and asked them to complete the anonymous online survey. Non-responders were sent up to three email reminders. The study was conducted in Switzerland at the Institute of Primary Health Care of the University of Bern (BIHAM) between September 2017 and April 2018. It was approved by the Ethics Committee of the Canton of Bern (reference number 2017–02188).

### Statistical analyses

First, we described GP characteristics by calculating proportions, means and standard deviations (SD). Next, we described the proportions of GPs deprescribing per case-vignette and per medication by calculating crude percentages and 95% confidence intervals (CI). We used McNemar’s test to compare cases with positive and negative history of CVD and calculated the mean number of medications GPs deprescribed. Then one author (SM) analysed the content of the GPs’ free text explanations for deprescribing medications. A senior author (SS) reviewed her codes and themes and helped finalize categories. Finally, we dichotomized Likert-scale responses to the questions in Part C into very important/important and reported as percentages. We analysed the content and coded responses to the final open-ended question. We defined a two-sided *p*-value of < 0.05 as significant. All analyses were performed with STATA 15.1 (StataCorp, College Station, TX, USA).

## Results

### GP characteristics

Of 282 GPs invited, 157 (56%) responded: 73% were men; mean age was 56 (SD 8); and, half the participants had > 25 years of experience in practice (Table 1). Most GPs (88%) estimated that they “frequently” or “very frequently” saw patients ≥70 years in their practice; 84% reported they “frequently” or “very frequently” considered deprescribing in their daily practice, but only 30% deprescribed “frequently” or “very frequently”.

**Table 1:** Baseline characteristics of participating GPs (*n* = 157)

Baseline characteristics	
Female, n (%)	42 (27)
Age, years (SD)	56 (8)
Experience as GP, years (SD)	20 (9)
Number of consultations on average per working day, n (%)	
< 15	12 (7)
15–25	67 (43)
26–35	61 (39)
> 35	17 (11)
How often do you see/treat patients > 70 with multimorbidity and polypharmacy? n (%)	
very rarely	1 (1)
rarely	3 (2)
occasionally	17 (11)
frequently	89 (57)
very frequently	46 (29)
How often do you deal with the topic of deprescribing medications in your daily practice? n (%)	
very rarely	0 (0)
rarely	0 (0)
occasionally	25 (16)
frequently	95 (61)
very frequently	36 (23)
How often do you deprescribe medications during consultations with your patients in your daily practice? n (%)	
very rarely	0 (0)
rarely	8 (5)
occasionally	101 (65)
frequently	39 (25)
very frequently	8 (5)

### Case-vignette analyses

In the case-vignette without CVD history, 153 GPs (98%) reported they would deprescribe at least one medication. On average, they would deprescribe 4.2 (95%CI 4.0–4.4) of the possible seven medications. In the case-vignette with CVD history, a similar proportion of GPs (97%) would deprescribe at least one medication; on average, they would deprescribe 3.3 (95%CI 3.1–3.6) medications (Table 2).

**Table 2:** Comparison of percentages of GPs reporting to deprescribe medication in the case of a frail 82-year-old patient without and with history of cardiovascular disease (CVD) and most frequently mentioned reasons to deprescribe for the case

	History of CVD				p-value^1^
	No		Yes		
Medication	Percentage of GPs (95% CI)	Reasons to deprescribe (frequency)	Percentage of GPs (95% CI)	Reasons to deprescribe (frequency)	
Atorvastatin 40 mg	100%	*- Not enough benefit (56)* *- No indication (35)* *- No evidence (16)* *- Short estimated life expectancy (16)*	76% (69–83%)	*- Not enough benefit (30)* *- Other (25)* *- Short estimated life expectancy (19)*	< 0.001
Pantoprazole 20 mg	88% (83–93%)	*- No indication (111)* *- In reserve, no long-term therapy (6)* *- Not enough benefit (5)*	81% (75–87%)	*- No indication (90)* *- Not enough benefit (7)*	0.002
Aspirin 100 mg	74% (67–81%)	*- No indication (55)* *- Not enough benefit (19)* *- Side effects (15)*	32% (25–40%)	*- Other (13)* *- Side effects (9)* *- Short life expectancy (8)*	< 0.001
Tramadol 50 mg	71% (63–78%)	*- Side effects (76)* *- Lower drug dose (8)* *- In reserve, no long-term therapy (8)*	70% (63–77%)	*- Side effects (69)* *- Lower drug dose (9)* *- In reserve, no long-term therapy (8)*	0.71
Amlodipine 5 mg	44% (36–52%)	*- No indication (22)* *- Side effects (22)* *- Not enough benefit (7)* *- Deprescribe the drug and evaluate the effect (7)*	36% (28–44%)	*- No indication (21)* *- Side effects (9)* *- Other (7)*	0.011
Paracetamol 1 g	29% (22–37%)	*- Lower drug dose (13)* *- In reserve, no long-term therapy (13)* *- Deprescribe the drug and evaluate the effect (6)*	29% (22–36%)	*- Lower drug dose (14)* *- In reserve, no long-term therapy (10)* *- Deprescribe the drug and evaluate the effect (6)*	0.56
Enalapril 10 mg	24% (17–31%)	*- No indication (15)* *- Side effects (5)* *- Lower drug dose (4)*	19% (13–25%)	*- No indication (13)* *- Lower drug dose (3)* *- Other (3)*	0.033

^1^P-value from McNemar’s test comparing percentages of GPs deprescribing each medication by CVD

In the case-vignette without history of CVD, reported willingness of participants to deprescribe was high for cardiovascular preventive medications like atorvastatin (100%) and aspirin (74%). Many GPs also reported that they would deprescribe at least one of the antihypertensive medications (44% selected amlodipine; 24% selected enalapril), and 88% would deprescribe pantoprazole. Far fewer GPs (29%) reported that they would deprescribe paracetamol.

When we compared the case-vignette with CVD history to the vignette without CVD, we found that 29% of GPs would deprescribe paracetamol in both cases (*p* = 0.56) and an almost equal percentage (70% vs. 71%, *p* = 0.71) would deprescribe tramadol. For patients with CVD history, a smaller percentage of GPs would deprescribe cardiovascular preventive medications like aspirin (32% vs. 74%, *p* < 0.001), atorvastatin (76% vs. 100%, p < 0.001), enalapril (19% vs. 24%, *p* = 0.033), amlodipine (36% vs. 44%, *p* = 0.011), and pantoprazole (81% vs. 88% *p* = 0.002).

### Reasons to deprescribe

When we categorized the reasons GPs gave for deprescribing in the frail oldest-old without CVD history we found they most frequently deprescribed aspirin for ‘no indication’ (36% of those who would deprescribe at least one medication), enalapril (10%), amlodipine (14%), and pantoprazole (73%). GPs gave ‘lack of benefit’ as the main reason for deprescribing atorvastatin (37%). ‘Side effects’ were the most common reason they would deprescribe tramadol (50%). They were less likely to deprescribe pain medication, especially paracetamol, than cardiovascular medication and explained that they would either reduce the dose (8%) or prescribe it as needed (8%).

GPs reasons for deprescribing for the frail oldest-old with CVD history were similar, except for atorvastatin and aspirin. For these drugs, GPs mentioned other reasons, including ‘no priority’, ‘not in >80 years’, and ‘not appropriate prevention’.

### Factors influencing deprescribing among GPs

GPs most commonly rated four factors as “important” or “very important” in their decisions about deprescribing: ‘risk of a medication’ (99%); ‘benefit of a medication’ (98%); ‘quality of life’ (98%); and, ‘life expectancy of the patient’ (96%) (Table 3). GPs considered the factors ‘expenditure of time for deprescribing’ (19%) and ‘self-dispensation of medication in GP office’ (which means in Switzerland drug delivery by GPs in their own office) (7%) to be much less important.

**Table 3:** Factors important to GPs (n = 157) when deprescribing (per GP more than one answer was possible)

Factors	Rated as very important or important, %
Risk of a medication	99%
Benefit of a medication	98%
Quality of life of the patient	98%
Life expectancy of the patient	96%
Potential negative health outcomes of medication’s change	76%
Interprofessional communication (between GPs and other prescribing physicians)	73%
Interprofessional collaboration (between GPs and other prescribing physicians)	72%
Age of the patient	73%
Existence of deprescribing guidelines	64%
Expectations of the patient	63%
Difficult communication (between GP and patient, e.g. due to cognitive impairment)	56%
Expectation of relatives	49%
Existence of tools that facilitate deprescribing	48%
Expenditure of time for thinking about and deprescribing in the older multimorbid patient with polypharmacy	19%
Self-dispensation of medication in GP office^1^	7%

^1^Self-dispensation means ‘drug delivery by general practitioners in their office’

In their response to the open-ended question, many GPs mentioned the importance of patients’ wishes and priorities, and that their own ‘assessment of cost/benefit of a medication’ and ‘drug interactions’ could influence their decision to deprescribe.

## Discussion

### Summary

In a hypothetical frail oldest-old patient on 7 long-term medications, GPs would deprescribe (cease or reduce the dose of) an average of 4 medications for patients with no CVD history and 3 medications for patients with CVD history. In either case, they would usually deprescribe cardiovascular preventive medication (statin, aspirin, blood pressure lowering medication) because they thought it lacked indication or benefit. They would retain pain medication, but might reduce it or prescribe it “as needed” if they expected side effects. Positive CVD history was associated with less deprescription of atorvastatin and aspirin, which may reflect the belief that potential risks like side effects outweighed potential benefits of cardiovascular preventive medication in patients with low CVD risk. This accords with the European guidelines on preventing cardiovascular disease, which recommend that patients with low CVD risk be given lifestyle advice and not necessarily treated with antihypertensives and/or lipid-lowering drugs [36, 37]. When they decided which medication to deprescribe, GPs considered the risk/benefit of the medication, and the patient’s quality of life and life expectancy to be important. They also took the views and priorities of their patient into account.

### Strengths and limitations

Our study had a higher-than-usual survey response rate (56% vs. the typical rate of 30–40%) [38] and our sample closely matches the general Swiss population of GPs in age, gender, and years in practice, but our results might not be generalizable to GPs in other countries where prescribing practices differ. The GPs we surveyed may have been more interested in deprescribing than GPs in the general population since our sample was taken from those who had already consented to participate in research studies. Our study was also limited by the deliberate simplicity of the case-vignettes we chose, since we were forced to omit potentially interesting patient and GP characteristics. For instance, the patient in the case-vignette has no chronic health problems and takes no medicines associated with adverse drug events or that pose a risk when deprescribed. But we deliberately chose this case-vignette for the following reasons: 1) to standardize the case; 2) to avoid overloading respondents with information, 3) to make participation more feasible for GPs, and 4) to ensure responders had a common understanding of the case. Our analysis relied on claims GPs made about their intentions (that they would deprescribe selected medications) but these may not reflect their true practice. To mitigate this problem, we used standardized case-vignettes to exemplify a complex problem frequently encountered by GPs who treat frail oldest-old patients with polypharmacy. A GP who intends to describe may find that they cannot follow through on the intention in clinical practice because they are faced with barriers and factors outside their control. Social desirability bias may also have affected our results; for example, only 19% of respondents reported that time was an important factor in deprescribing, though the qualitative literature regularly reports time as a significant barrier to deprescribing [20, 39]. But the anonymous nature of the survey may have minimised this bias. Our case-vignette may also have encouraged the GP to opt to deprescribe, since the patient has impaired physical function and is likely to have limited life expectancy. In the vignette, the patient’s MMSE was 12 and he depended on others for activities of daily living. Since dementia does not progress predictably, and varies between individuals, we could describe a patient with limited life expectancy, but not be any more specific. If we told the GP the patient was expected to live less than 12 months or needed palliative care, it might have changed our results [40, 41].

### Comparison with existing literature

Our quantitative research complements qualitative findings by *Sinnige* et al. [42]*,* who assessed GPs’ medication management strategy and factors that influenced the deprescribing process in a similar setting. They also used case-vignettes for hypothetical patients to understand how GPs would deprescribe, identified patient- and medication-related factors that influenced medication management and highlighted the importance of taking a patient-centred approach, considering the patient’s age and life expectancy, and weighing patient’s preferences and perspectives into the decision.

Our study accords with previous research that showed CVD history influences GP prescribing decisions [33, 36]. We found higher rates of deprescribing statins than did previous studies [43], perhaps because the patient in our case-vignette was a nursing home resident. Deprescribing patterns might have been different in patients with no or mild cognitive impairment [44]. Our case-vignette also provided sparse information about family and caregiver involvement and advanced directives might have facilitated deprescribing.

Ní Chróinín et al. [45] also used case-vignettes in a similar study of deprescription among geriatricians (*N* = 930, response rate 14,4%). Like Ní Chróinín et al., we found considerable willingness to deprescribe cardiovascular preventive medication in the scenario of cognitive impairment and dependency. Ní Chróinín et al.’s sample included a higher percentage of women and younger geriatricians than are present in the general population of GPs. Our study population more closely matches the GP population in age, gender, and years in practice, so our results suggest these observations are more generally applicable.

Patients with dementia may be undertreated for pain [46, 47], possibly because members of this group express pain differently than those without dementia (particularly if patients with dementia are non-verbal) [48]. Pain symptoms like agitation or aggression may be attributed to dementia (labelled behavioural and psychological symptoms of dementia) and not treated appropriately [49]. It is thus unsurprising that GPs were less likely to deprescribe pain medications, particularly paracetamol, than cardiovascular medications. Since under-treatment of pain is a concern in people with dementia, the proportion of GPs who would deprescribe paracetamol (29%) when an individual has chronic back pain may be higher than ideal, but 13/19 of the GPs we surveyed would prescribe paracetamol as a reserve medication instead of eliminating it entirely. A high proportion of GPs reported they would deprescribe tramadol, perhaps because of the risks the medication poses. Our study was not designed to determine which medications GPs would prescribe to best manage the patient’s pain (for example, starting oxycodone instead of tramadol or initiating non-pharmacological management). GP’s deprescribing patterns may also have been influenced by the results of recent studies that found paracetamol and opioids might not be effect for treating chronic pain [50, 51].

We found GPs heavily weighted the patient’s quality of life and life expectancy, wishes and priorities, in their decisions about deprescribing, perhaps because the patient in the case vignette had advanced dementia. A focus on quality of life is a key part of modern medical care for people with dementia [52]. Our findings complement those of recently published reviews of patient barriers to and enablers of deprescribing [53, 54] which emphasize the importance of centring the deprescribing process on the patient [55]. Elements of patient-centred care include shared decision making, viewing the person as a whole, and maintaining a positive doctor-patient relationship [56]. But our study was not designed to determine whether GPs felt able to share decision-making about deprescribing, or how they approached the discussion with patients. Other studies found that GPs would appreciate guidelines or tools that made it easier for them to deprescribe [14, 22, 39, 57]. The Swiss GPs we included in our studies would welcome this but did not prioritise it. Our findings also dovetail with results from qualitative studies that assessed why GPs decide to deprescribe [20]. Our research suggests that Swiss GPs would try to reduce medication burden in frail individuals with multimorbidity and polypharmacy through deprescribing. However, while most responded that they regularly dealt with the topic of deprescribing in practice, only 30% reported that they frequently deprescribed.

### Implications for research and practice

We did not assess barriers to deprescribing (like fear of negative consequences) in our case-vignette. Further clinical trials are needed to measure the safety, benefits, and best practices for deprescribing, especially in oldest-old multimorbid patients. We also suggest researchers explore more complex cases in the future by adding details to case-vignettes. They may also wish to ask GPs about deprescribing in a stable patient without current problems, to see if it changes the results. We hope others will explore the reasons GPs prioritise or do not prioritise reviewing medicines with an eye to deprescribing. Since medication and patient characteristics are important factors in deprescribing, researchers should also study patient and family beliefs and attitudes. If we knew more about how, why, and when GPs decide to deprescribe, it should be possible to develop tools that assist them in balancing these (sometime competing) interests.

## Conclusion

In case-vignettes, Swiss GPs were most likely to deprescribe cardiovascular preventive medication, citing lack of indication and benefit, and less likely to deprescribe pain medications. Overall, Swiss GPs expressed willingness to deprescribe for frail oldest-old patients and were guided in their decisions by the risks and benefits of a medication, quality of life and life expectancy of patients, and patient priorities.

## Additional file

Additional file 1: The survey used in the LESS Study. (DOCX 21 kb)

## Abbreviations

ADE: Adverse drug event
Bid: Twice daily
CI: Confidence interval
CVD: Cardiovascular disease
GP: General practitioner
Od: Once daily
OR: Odds ratio
PPI: Proton-pump inhibitor
SD: Standard deviation
Tid: Three times daily
Vs: Versus
