# Supplementary material for: How general practitioners would deprescribe in frail oldest-old with polypharmacy — the LESS study
Source: BMC Fam Pract. 2018 Oct 12;19:169. doi: 10.1186/s12875-018-0856-9 (PMC6186124; doi:10.1186/s12875-018-0856-9)
Supplement: Supplementary file 1 — The survey used in the LESS Study. (DOCX 21 kb) [file 12875_2018_856_MOESM1_ESM.docx]

**Additional File 1**. The survey used in the LESS Study

**Barriers and Enablers to the Willingness to Deprescribe 
The LESS Study for General Practitioners**

**A) GP background information**

1. Please indicate your age (in number of years).

2. Please indicate your gender. (male/female)

3. How many years have you been working as GP? (in number of years)

4. How many clinical consultations do you have on average per working day? (An average working day is a full day/2 sessions in the practice). (<15, 15-25, 26-35, >35)

5. How often do you see/treat patients who fulfil the following criteria:

- aged ≥70 years
- ≥3 chronic conditions
- ≥5 regular medications

(never, rarely, occasionally, frequently, very frequently)

Thinking of the patients who fulfil these three criteria. How would you answer the following questions?

- aged ≥70 years
- ≥3 chronic conditions
- ≥5 regular medications
- 6. How often do you deal with the topic of deprescribing medications in your daily practice with these patients? (never, rarely, occasionally, frequently, very frequently)
- 7. How often do you deprescribe medications during consultations with your patients in your daily practice in respect of these patients? (never, rarely, occasionally, frequently, very frequently)

**B) Case-vignette**

In the following, there will be a case-vignette.

After the case-vignette, there will be a few questions asking you which medications you would deprescribe.

**Patient X**, 82 years of age:

**Social history**: retired carpenter*, lives together with his wife in a nursing home*

**General health**: *Patient X walks very little using a walker. Needs daily support for personal hygiene and getting dressed/undressed. Lack of spatial or temporal orientation. Unintended weight loss of 8kg in the past two months. MMSE 12/30.*

**Other diagnoses**: Chronic back pain, hypertension (last blood pressure measurements ranged from 130 to 140mmHG, systolic), non-smoker, no family history of cardiovascular events

**Laboratory values**: Dyslipidemia (LDL 3,8mmol/l), liver and kidney function are normal (taking into account the age of the patient), normal blood count

**Daily medication intake:**

Aspirin 100 mg once daily

Atorvastatin 40 mg once daily

Enalapril 10 mg once daily

Amlodipine 5 mg once daily

Paracetamol 1 g three times a day

Tramadol 50 mg twice daily

Pantoprazol 20mg once daily

**In this case-vignette, you consider the patient:**

- to have strongly impaired physical functioning

- to be strongly dependent in his daily routine

- to be cognitively strongly impaired

- to have a low risk of cardiovascular events

- 16. Would you deprescribe or decrease the dosage of one/several medication/s? (yes/no)
- 17. Which medication/s would you deprescribe or decrease?
  - Aspirin 100 mg once daily
  - Atorvastatin 40 mg once daily
  - Enalapril 10 mg once daily
  - Amlodipine 5 mg twice daily
  - Paracetamol 1g three times a day
  - Tramadol 50 mg twice daily
  - Pantoprazole 20 mg once daily
- 18. Consider that this Patient had a cardiovascular event in the past (e.g. myocardial infarction three years ago). Would you deprescribe or decrease the dosage of one/several medication/s? (yes/no)
- 19. Which medication/s would you deprescribe or decrease taking into account that Patient X had a cardiovascular event in the past (e.g. myocardial infarction three years ago)?
  - Aspirin 100 mg once daily
  - Atorvastatin 40 mg once daily
  - Enalapril 10 mg once daily
  - Amlodipine 5 mg twice daily
  - Paracetamol 1g three times a day
  - Tramadol 50 mg twice daily
  - Pantoprazol 20 mg once daily

**C) Barriers and enablers to the willingness to deprescribe**

20. How important are the following ***patient characteristics*** for you when you deprescribe medications?

|  | Not important | Slightly important | Neutral | Important | Very important |
| --- | --- | --- | --- | --- | --- |
| Age |  |  |  |  |  |
| Life expectancy |  |  |  |  |  |
| Quality of life |  |  |  |  |  |
| Previous experiences with deprescribing |  |  |  |  |  |
| Expectations of the patient |  |  |  |  |  |
| Potential negative health outcomes |  |  |  |  |  |
| Difficult communication |  |  |  |  |  |
| Expectation of relatives |  |  |  |  |  |

21. How important are the following criteria for you when you deprescribe medications?

|  | Not important | Slightly important | Neutral | Important | Very important |
| --- | --- | --- | --- | --- | --- |
| Existence of deprescribing guidelines |  |  |  |  |  |
| Existence of tools that facilitate deprescribing |  |  |  |  |  |
| Interprofessional communication (between GPs and other prescribing physicians) |  |  |  |  |  |
| Interprofessional collaboration (between GPs and other prescribing physicians) |  |  |  |  |  |
| Expenditure of time |  |  |  |  |  |
| Self-dispensation of medication in GP office |  |  |  |  |  |
| Benefit of a medication |  |  |  |  |  |
| Risk of a medication |  |  |  |  |  |

22. Are there any other factors that influence deprescribing from your point of view? (yes/no)

In your opinion, which other factors influence deprescribing?

**Remarks and comments**

23. Do you have any additional comments or remarks regarding deprescribing?
